# Cellular senescence: Neither irreversible nor reversible

**DOI:** 10.1084/jem.20232136

**Published:** 2024-02-22

**Authors:** Maurice Reimann, Soyoung Lee, Clemens A. Schmitt

**Affiliations:** 1 Medical Department of Hematology, Oncology and Tumor Immunology, Corporate Member of Freie Universität Berlin, Humboldt-Universität zu Berlin, and Berlin Institute of Health, and Molekulares Krebsforschungszentrum—MKFZ, Campus Virchow Klinikum, Charité—Universitätsmedizin, Berlin, Germany; 2 Johannes Kepler University, Linz, Austria; 3 Department of Hematology and Oncology, Kepler University Hospital, Linz, Austria; 4 Max-Delbrück-Center for Molecular Medicine in the Helmholtz Association, Berlin, Germany

## Abstract

Cellular senescence is a critical stress response program implicated in embryonic development, wound healing, aging, and immunity, and it backs up apoptosis as an ultimate cell-cycle exit mechanism. In analogy to replicative exhaustion of telomere-eroded cells, premature types of senescence—referring to oncogene-, therapy-, or virus-induced senescence—are widely considered irreversible growth arrest states as well. We discuss here that entry into full-featured senescence is not necessarily a permanent endpoint, but dependent on essential maintenance components, potentially transient. Unlike a binary state switch, we view senescence with its extensive epigenomic reorganization, profound cytomorphological remodeling, and distinctive metabolic rewiring rather as a journey toward a full-featured arrest condition of variable strength and depth. Senescence-underlying maintenance-essential molecular mechanisms may allow cell-cycle reentry if not continuously provided. Importantly, senescent cells that resumed proliferation fundamentally differ from those that never entered senescence, and hence would not reflect a reversion but a dynamic progression to a post-senescent state that comes with distinct functional and clinically relevant ramifications.

## Teaser

Cellular senescence is a stress-inducible terminal cell-cycle arrest program with far-reaching pathophysiologic implications. This Review summarizes the evidence that senescent cells need to actively maintain their arrest machinery and discusses the consequences if these epigenome-remodeled cells resume proliferation in a “post-senescent” condition.

## Introduction

Seminal observations by Hayflick and Moorhead first described the limited proliferative lifespan normal cells exhibit in culture (Hayflick and Moorhead, 1961), subsequently, mechanistically underscored by the demonstration that progressive shortening and critical erosion of telomeres result in a terminal growth arrest (Bodnar et al., 1998; Yu et al., 1990). This condition, also known as replicative senescence (RS), has been considered irreversible if not blocked by ectopic overexpression of the telomerase reverse transcriptase protein TERT (Bodnar et al., 1998). Importantly, senescent cells accumulate with age in vivo (Herbig et al., 2006), indicating that their occurrence represents a potential link between cellular and organismic aging and is not merely a cell culture-related phenomenon.

**Figure 1.**
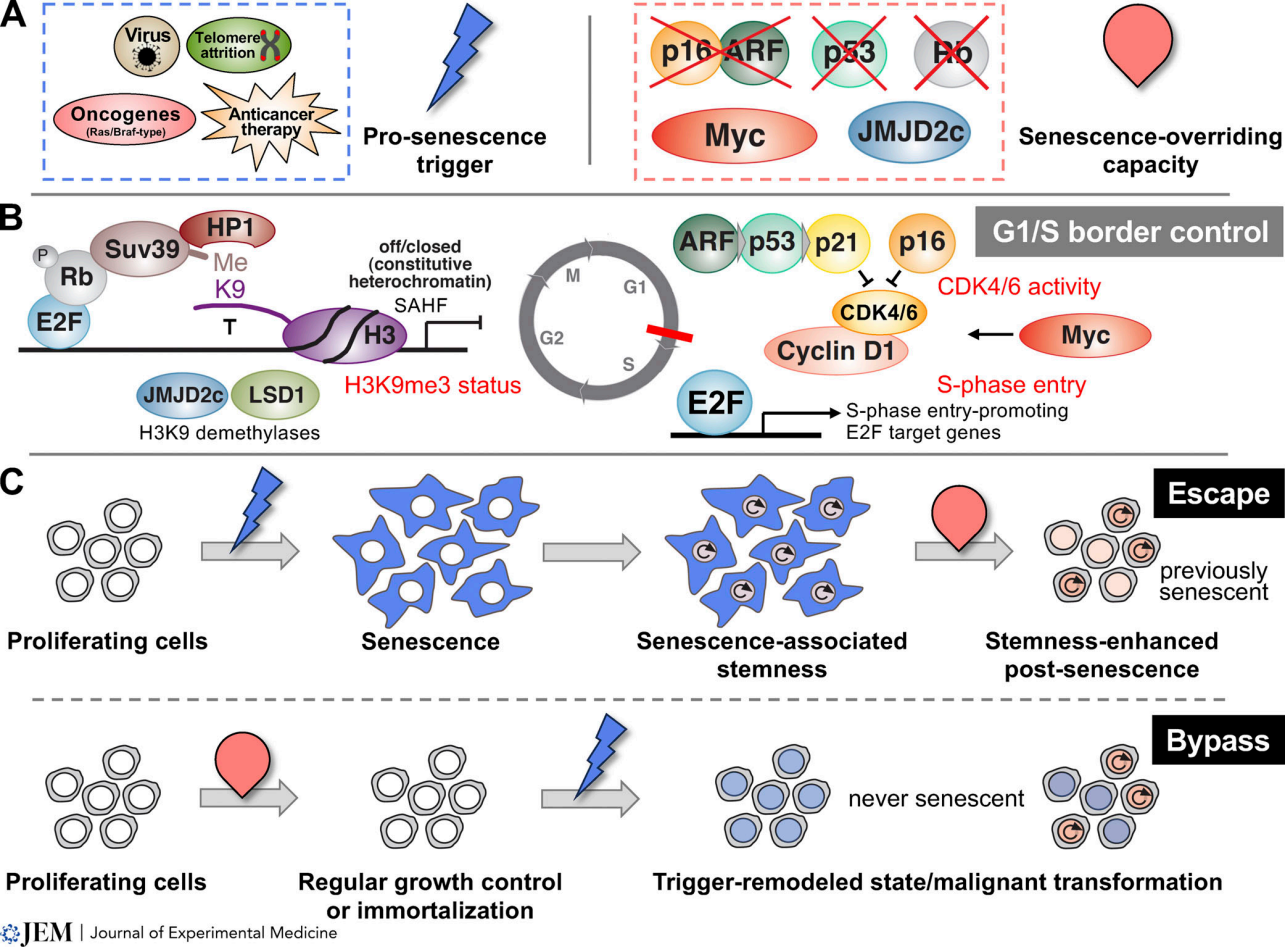
**Senescence escape versus bypass. (A)** A variety of pro-senescent triggers (blue bolt) that account for RS, OIS, TIS, or VIS, respectively (left), and senescence-overriding capacities (red oval) that disable the cellular capacity to respond to pro-senescent triggers (as in A) with senescence entry, or promote cell-cycle reentry out of manifest senescence (right). Specific examples include lost expression of the two gene products p16^INK4a^ and p14/p19^ARF^ (the latter operating as a p53 upstream activator) encoded by the *CDKN2A* locus (compromised by mutations, deletions, and/or promoter hypermethylation), p53 inactivation (typically by missense mutations and/or allelic deletion), Rb inactivation (due to mutations or deletions), Myc overexpression (in conjunction with Ras/Braf type oncogenes), or overexpression of histon H3-lysine 9 (H3K9)-active demethylases (such as JMJD2c or LSD1). **(B)** Transcriptionally repressive H3K9me3-decorated senescence-associated heterochromatin foci (SAHF) formation in the vicinity of S-phase entry-promoting E2F-driven target gene promoters upon recruitment of an H3K9 methyltransferase capacity (such as Suv39h1) that binds in conjunction with heterochromatin protein 1 (HP1) to G1-phase-typical hypophosphorylated Rb protein complexed to E2F transcription factors, thereby firmly blocking the cell in G1 (left). Additional components of the G1/S border control are cyclin-dependent kinase (CDK) four and six inhibitors p16^INK4a^ and p21^CIP1^, the latter a p53 target gene, that counter CDK4/6-cyclin D1-mediated cell-cycle progression (right). Senescence-specific upstream activation of ARF/p53 and p16^INK4a^ is not entirely clear but involves DNA damage signaling, FoxO transcriptions factors, and the MAPK/ETS cascade (not shown). Key barriers to cell-cycle reentry out of senescence are highlighted in red (and can be overridden by the indicated gene moieties): the H3K9me3 status (disrupted by elevated H3K9 demethylase activities such as JMJD2c or LSD1), repressed CDK4/6 activity (de-repressed by CDK4 amplification and/or inhibitor-insensitive mutations such as CDK4-R24C, or reduced p16^INK4a^ or p21^CIP1^ inhibitor expression), or enforced S-phase entry (e.g., via Myc overexpression). Notably, inducible gene moieties—e.g., a doxycycline-controlled p53-targeting small-hairpin RNAs or a 4-OHT-responsive JMJD2C:ER^TAM^ fusion—were successfully experimentally employed to enforce a senescence exit. **(C)** Distinct cellular journeys in which pro-senescent triggers were encountered by senescence-capable versus a priori senescence-incapable cells. Senescence-capable cells respond to pro-senescent triggers with senescence entry, typically associated with wound healing-reminiscent reprogramming into a latent transcriptional stem-like state (e.g., elevated Wnt and Notch signaling). Cells may experience senescence-overriding gene alterations (i.e., either overexpression of senescence-disabling or lost expression of senescence maintenance-essential gene moieties as outlined in A and B) while being in senescence (e.g., due to DNA replication-independent *CDKN2A* promoter hypermethylation or an inability to continuously reestablish repressive H3K9me3 marks [which are subject to nucleosome turnover] at proliferation-promoting target gene promoters). These senescent cells with their senescence-associated stemness may resume proliferation (i.e., escape) out of senescence with particularly aggressive growth properties as “post-senescent” or “previously senescent” cells due to retained marks of senescence-associated epigenomic remodeling (top). In contrast, exposure of cells with an a priori senescence defect (as outlined in A and B, potentially resulting in immortalization, i.e., the ability to divide indefinitely) to a pro-senescent trigger will not lead to senescence and associated epigenomic remodeling, hence will bypass senescence and produce trigger-specific remodeled or even -transformed cellular conditions without a history in senescence (“never senescent”; bottom). Note that neither escape nor bypass cells appear to exhibit growth properties similar to their proliferating ancestors; especially escape cells are no mere senescence revertants but distinctly different from their pre-senescent counterparts. See main text for additional details and references.

Types of senescence that are more acutely or “prematurely” evoked by cellular insults such as oncogenic activation, anticancer therapy, or viral infection, leading to oncogene-induced senescence (OIS), therapy-induced senescence (TIS), or virus-induced senescence (VIS), respectively, are largely indistinguishable from RS-like phenotypes including a lastingly stable growth arrest (Fig. 1 A). Therefore, the field readily extrapolated the RS-based assumption of an irreversible cell-cycle block to other forms of senescence. Like eroded telomeres inducing RS, activated Ras/Braf oncogenes, the prototypic drivers of OIS, continue to signal throughout the remaining lifetime of the affected cells. However, triggers of TIS or VIS are not necessarily permanent, although they may also account for chronic, difficult-to-repair DNA damage, for instance at telomeric sites (Fumagalli et al., 2012; Rodier et al., 2011), continuously entertaining a DNA damage response (DDR). Notably, the duration and quality of such prosenescent trigger to maintain the senescent state (see below) are likely to vary across different senescence types, especially when senescence associated–secretome (a.k.a., senescence-associated secretory phenotype [SASP])-mediated paracrine (or secondary) senescence, as well as T-helper cell cytokine-induced senescence (Acosta et al., 2013; Braumüller et al., 2013) are considered. Indeed, experimental evidence of truly long term–arrested cells under adequate cell culture settings outside telomere-initiated RS is largely missing (Fumagalli et al., 2014), and the mere detectability of senescent cells in vivo cannot provide insights into the prehistory of these cells. Moreover, lastingly arrested cells may be lost in vivo due to immune clearance, thereby further complicating the assessment of senescence stability (Chen et al., 2023a; Eggert et al., 2016; Kang et al., 2011; Marin et al., 2023; Reimann et al., 2021; Xue et al., 2007).

Senescence naturally emerged as a cellular condition to fulfill certain physiologic purposes. The functional designation of senescent cells must be viewed in their specific contextual needs, neither likely to be an indefinite task nor an “eternal” determination. Senescent cells play numerous roles in complex, dynamic processes such as embryonic development, where the developmental stage of one compartment instructs a neighboring compartment or wound healing, where a coordinated sequence of protective, clearing, and regenerative steps is needed to defend microbial intruders, eliminate debris, and ensure fully regained parenchymal functionality (Demaria et al., 2014; Lee and Schmitt, 2019; Muñoz-Espín et al., 2013; Ritschka et al., 2017; Storer et al., 2013). These processes have in common that both cell-autonomous and non-cell-autonomous (i.e., SASP) features of senescent cells are cooperatively needed, that senescent cells—in various ways—contribute to tissue homeostasis and regeneration, and that their role is not to persist indefinitely.

In essence, despite the widespread perception of cellular senescence as an endpoint response to a variety of triggers, the notion of its irreversible nature is poorly substantiated. Especially the irrevocable growth arrest, although widely referred to in original research publications and review articles on premature types of senescence, builds on the analogy to RS rather than robust evidence for a long-lasting cell-cycle block. Notably, the definitive judgment of a condition as irreversible is beyond experimental proof, rendering it, to some extent, a semantic or a philosophic problem.

## Thought to be irreversible

Prematurely senescent cells in OIS, TIS, and VIS seem to employ similar signaling cascades that underlie the limited division potential, the “mitotic clock,” of genetically unaltered cells in RS (Schmitt et al., 2022, 2023). In contrast, immortalized cells such as those lacking functional p53 or those overexpressing a p16^INK4a^-overriding cyclin-dependent kinase (CDK) moiety such as CDK4-R24C (Wölfel et al., 1995) can tolerate prosenescent signals from eroded telomeres or other lasting cellular insults and may indefinitely divide (Harvey and Levine, 1991; Herbig et al., 2004; Rane et al., 2002) (Fig. 1 A).

Cellular senescence typically reflects a firm proliferative arrest in the G1 phase of the cell cycle. In cycling cells, hyperphosphorylation of the retinoblastoma (Rb) protein at the end of the G1 phase releases E2F transcription factors (TF) from their binding to Rb, thereby enabling them to promote S-phase entry through activation of their target genes. Under prosenescent stimuli—though the precise molecular mechanism is yet to be elucidated—the intact Rb–E2F complex recruits histone methyltransferases such as Suv39h1, which decorate E2F promoters in their vicinity with transcriptionally repressive lysine-9-trimethylated histone H3 (H3K9me3) marks. Collectively, these marks form senescence-associated heterochromatin foci (SAHF) as a stably arrest-conferring epigenetic mechanism that is insensitive to external growth stimuli (Braig et al., 2005; Narita et al., 2003) (Fig. 1 B). Beyond the SAHF, detectable only in a subset of mostly human senescent cells, cellular senescence is generally characterized by a number of morphological, biochemical, and functional vignettes, which are typically used in conjunction to assign a cell as being senescent or not.

Detection of senescent cells in culture and tissues is central to studying their role in health and disease, but it has been notoriously difficult. Unfortunately, no single marker has emerged to faithfully and unequivocally determine the senescent status of a cell. Depending on biological material and experimental settings, a panel of several markers, ideally including the gold-standard senescence-associated β-galactosidase (SA-β-gal) assay (Dimri et al., 1995), reflecting enhanced lysosomal activity, or detectability of the lysosomal “age pigment” lipofuscin, cell division indicators such as Ki-67 or carboxyfluorescein diacetate succinimidyl ester (CFSE)-based membrane labeling, expression analyses of CDK inhibitors p16^INK4a^ or p21^CIP1^ (Lee et al., 2021), the senescence-associated secretory phenotype (SASP [Coppé et al., 2008]), and other senescence-related gene sets, as well as actual growth assessment, is needed to robustly diagnose the senescent status in vitro, or, even more challenging, in vivo (Gorgoulis et al., 2019; Schmitt et al., 2022) (Box 1). A key limitation regarding the practical applicability of senescence-related markers is the need for their codetection within the same cell to compellingly call the status of such cell senescent. Any kind of bulk analysis can only serve as an approximation of the senescent cell content in an entire cell population. Bulk analyses may be informative when conducted in homogeneous cell populations, e.g., human diploid fibroblasts, in which the synchronized activation of a pro-senescent oncogene triggers OIS in the vast majority of the cells (Yu et al., 2018). Despite the increasing number of multigene-based signatures, typically presented as a large panel of senescence-associated and expression-quantified transcripts such as the “Suvarness” signature (Schleich et al., 2020), SENESCOpedia (Jochems et al., 2021), SenMayo (Saul et al., 2022), or SenPred (Hughes et al., 2023a, *Preprint*), verification of their value in different tissues and settings is needed. Multiplexed single-cell technologies that simultaneously detect protein- or transcript-based senescence marker panels overcome diagnostic limitations in heterogeneous populations of cells assayed in vitro or after cell singularization from biopsies ex vivo (SenNet Consortium, 2022; Gurkar et al., 2023; Yuan et al., 2019). They may also integrate morphological information and increasingly incorporate machine learning–based methods (Belhadj et al., 2023; Heckenbach et al., 2022; Hughes et al., 2023b; Kusumoto et al., 2021; Wallis et al., 2022). Immunohistochemical staining of serial tissue sections with multiple antibodies is widely used as an approximation to assign numerous markers to the same cells but is technically not fully satisfying. With the advent of multiplex protein- and transcript-based spatial single-cell omics, accurate designation of cells in their natural environmental context as being senescent or not is now within reach and will overcome the current difficulties of marking senescent cells in vivo (SenNet Consortium, 2022; Gurkar et al., 2023). Nevertheless, no marker panel has been identified so far that would work as a universal diagnostic tool—given the cell- and context-dependent differences that apply to the molecular presentation of senescent cells across organs and conditions.

Box 1 Cellular markers used as an approximation to diagnose senescence

• Senescence-associated β-galactosidase (SA-β-gal) activity staining using X-gal or its fluorescent variations as viable non-endpoint assays based on 5-dodecanoylaminofluorescein-di-β-d-galactopyranoside or SPiDER-β-gal as substrates (Dimri et al., 1995; Doura et al., 2016).
• Lysosomal “age pigment” lipofuscin staining using the Sudan Black-B-based GL13 (SenTraGor) assay (Lee et al., 2021; Rizou et al., 2019) or a fluorescence-conjugated Sudan Black-B analog thereof, termed GLF16 (Magkouta et al., 2023).
• Growth curve analysis (Braig et al., 2005; Serrano et al., 1997; Yu et al., 2018).
• Proliferation marker Ki-67 immunostaining (Haugstetter et al., 2010; Reimann et al., 2010).
• Lack of 5′-bromo-2′-deoxyuridine (BrdU), alternatively 5′-ethynyl-2′-deoxyuridine (EdU), incorporation (in vitro or in animal models in vivo) (Dörr et al., 2013; Milanovic et al., 2018; Reimann et al., 2010).
• Characteristic light scatter alterations by flow cytometry (regarding increased cell size and enhanced granularity) (Braig et al., 2005).
• Characteristic ultrastructural alterations by electron microscopy (Dörr et al., 2013; Narita et al., 2003).
• Carboxyfluorescein diacetate succinimidyl ester (CFSE)-based membrane labeling (to recognize proliferating cells by half of their fluorescence intensity after every division) (Milanovic et al., 2018).
• γ-H2AX immunostaining to mark DNA damage foci (Bartkova et al., 2006).
• p16^INK4a^ (CDK4/6 activity-blocking cell-cycle inhibitor) by RQ-PCR and immunostaining (Lee et al., 2021; Schmitt et al., 2002; Serrano et al., 1997).
• p21^CIP1^ (CDK activity-blocking cell-cycle inhibitor) by RQ-PCR immunostaining (Lee et al., 2021).
• p19^ARF^ (positive upstream regulator of p53) by RQ-PCR immunostaining (Schmitt et al., 2002).
• p53 and phospho-53-Ser15 (Bartkova et al., 2006; Schmitt et al., 2002).
• H3K9me3 immunostaining, flow cytometry, or chromatin immunoprecipitation to detect E2F target gene promoters (Narita et al., 2003; Reimann et al., 2010; Yu et al., 2018).
• HP1-γ immunostaining (to detect an H3K9me3 binding protein) (Braig et al., 2005; Haugstetter et al., 2010).
• 4′,6-Diamidino-2-phenylindole (DAPI)-dense senescence-associated heterochromatin foci (SAHF) (Narita et al., 2003; Yu et al., 2018).
• Phospho-ERK immunostaining (to detect activated MAPK signaling) (Haugstetter et al., 2010).
• SASP factors (Coppé et al., 2008) by RQ-PCR, multiplex protein detection, or, e.g., IL-8 or PAI-1 by immunostaining (Haugstetter et al., 2010; Lee et al., 2021).
• Associated stem cell markers, e.g., aldehyde dehydrogenase activity by flow cytometry or Wnt signaling (e.g., nuclear β-catenin by immunofluorescence) (Milanovic et al., 2018).

Senescent cells are heterogeneous in many ways—between individuals, across tissues, and even within the affected cell population in response to the same trigger. Interindividual differences in the propensity to senesce, and on senescence depth, quality, and stability might reflect a critical personal rheostat for aging, chronic inflammatory responses, and, ultimately, cancer risk. Beyond the depth and dynamics (see below) of the senescent condition, a cell population collectively exposed to a senescence trigger is unlikely to respond with a uniform marker profile. For instance, mosaic patterns for the CDK inhibitors p16^INK4a^ or p21^CIP1^ have been described in Braf-V600E-senescent melanocytes (Michaloglou et al., 2005; Pakuła et al., 2020). Moreover, reports on “senescence-like” presentations make it even more difficult to draw the line between “real” but heterogeneous senescence, aberrant forms of senescence, and “look-alike” states. A prominent example in this regard is cells with dysfunctional p53: *p53*^*−/−*^ cells, wild-type cells transduced with a p53-inhibiting peptide, or cells expressing a dominant-negative p53 mutant have been shown to disable “classic” G1-phase-arrested cellular senescence (Beauséjour et al., 2003; Milanovic et al., 2018; Serrano et al., 1997). However, SA-β-gal-positive senescence of p53-mutant cells is frequently mentioned in the literature because they typically arrest in the G2/M-phase; its molecular overlap with the classic G1-halted type remains to be characterized in greater depth (Nayak et al., 2017).

Despite increasing insights into alternative long-term arrest phenotypes such as quiescence, dormancy, hibernation, torpor, or diapause-like states, clearly discriminating or overlapping features, including the possibility of floating changes of individual cells, between these conditions are hard to define, and misinterpretations are likely to occur (Batlle and Clevers, 2017; Beauséjour et al., 2003; Bouma et al., 2012; Dias et al., 2021; Endo and Inoue, 2019; Ng et al., 2016; Oedekoven et al., 2021; Peeper et al., 2001; Phan and Croucher, 2020; Sage et al., 2000; Sang et al., 2008; Triana-Martínez et al., 2020) (see Table 1 for a more detailed comparison of long-term arrest phenotypes). Briefly, senescent cells are firmly arrested, metabolically highly active, and typically insensitive to classic external or internal mitogenic signals (He and Sharpless, 2017; Herranz and Gil, 2018; Lee and Schmitt, 2019), while dormant cells reflect an evolutionary adaptation with reduced metabolic activity to an unsuitable ecological environment but retained susceptibility to external growth-promoting triggers (Endo and Inoue, 2019; Phan and Croucher, 2020; Triana-Martínez et al., 2020). Quiescent cells are not-yet-dividing, metabolically economized cells that lack environmental stimuli—i.e., growth factors and nutrients—to unfold their intrinsic growth potential (Batlle and Clevers, 2017; Ng et al., 2016). Given the varying firmness of the cell-cycle arrest attributed to all of these conditions and the lack of discriminative markers that would confirm their distinctiveness, a truly irreversible growth cessation is unlikely to apply solely to senescence but not to the other states.

**Table 1.** Principles and characteristics of lasting cell-cycle arrest conditions

Long-term arrest condition	Senescence	Quiescence	Dormancy^a^	Diapause-like
Features				
*Lead biological property*	Terminal cell-cycle arrest and secretion (SASP) (Herranz and Gil, 2018; Schmitt et al., 2023)	Stand-by arrest under insufficient growth-supportive conditions (Marescal and Cheeseman, 2020)	Protective, hibernation-like economized survival strategy, likely overlapping with quiescence—possibly as the “quiescence of stem-like cells” (Triana-Martínez et al., 2020)	A state of suspended development as a reproductive survival strategy under unfavorable environmental conditions, especially insufficient nutrient supply (originally leading to delayed blastocyst implantation but adopted by other cells as a diapause-like adaptation) (Hu et al., 2020)
*Biomedical implications*	Embryonic development, wound healing, natural aging versus age-related pathologies, cancer development and therapy, auto-immunity, cardiovascular disorders, metabolic diseases, neurodegeneration, and virus infection (Baker et al., 2011, 2016; Bodnar et al., 1998; Budamagunta et al., 2021; Bussian et al., 2018; Demaria et al., 2014; Gorgoulis et al., 2019; Hayflick and Moorhead, 1961; Lee and Schmitt, 2019; Lee et al., 2021; McHugh and Gil, 2018; Muñoz-Espín et al., 2013; Schmitt et al., 2023; Song et al., 2020; Storer et al., 2013; Yu et al., 1990)	Reduced mitochondrial activity to protect from oxidative damage (Marescal and Cheeseman, 2020)	Protective low-level metabolic state in less supportive environment, reversible upon changes of external conditions—hence, an adaptive survival mechanism, deleterious as a cancer cell persister state (difficult to target and a risk as a source of late recurrence or metastasis), latent pluripotency program (Endo and Inoue, 2019; Phan and Croucher, 2020; Triana-Martínez et al., 2020)	As a “diapause-like” state usurpation of an embryonic program to lower both nutritive needs and cellular vulnerabilities under ongoing stresses (such as anticancer therapy) (Dhimolea et al., 2021; Hu et al., 2020)
*Impact on tumor fate*	Tumor-suppressive (acute) and tumor-promoting (via SASP and long-term persisters), the potential similarity between long-term persistent senescent cells and dormant cells, epithelial–mesenchymal transition (EMT) (Ansieau et al., 2008; Schmitt et al., 2023; Triana-Martínez et al., 2020)	As a mere quiescent state presumably tumor-suppressive, but less treatment-sensitive, see also dormancy or senescence	Tumor-suppressive (even of oncogenic signaling), but a potential source of late relapses, especially metastasis (arising from early disseminated cancer cells), partial EMT features (Harper et al., 2016; Riethmüller and Klein, 2001; Triana-Martínez et al., 2020)	Similar to a drug-tolerant persister state, diapause-like high signature-positive colorectal cancer patients experience inferior outcome (Takata et al., 1998)
*Mechanisms of arrest control*	Eroded telomeres, mitogenic oncogenes, anticancer therapeutics, virus infection and pro-senescent cytokines as triggers, PTEN loss, CDK inhibition, cooperation of upstream damage signaling (replication stress, DNA damage), elevated cell-cycle inhibitor expression and heterochromatinization of growth-promoting gene loci; SASP-mediated paracrine senescence as a reinforcing mechanism (Acosta et al., 2013; Alimonti et al., 2010; Bartkova et al., 2006; Braumüller et al., 2013; Coppé et al., 2008; Di Micco et al., 2006; Narita et al., 2003; Perez et al., 2015; Reimann et al., 2010)	Insufficient supply of external growth signals, niche signals, and/or nutrients, progression to a firmer senescent arrest might be prevented by the transcriptional repressor HES1 (Sang et al., 2008)	Induced by less supportive microenvironmental cues (e.g., hypoxic regions), “seed & soil” imbalance-driven, deprivation of growth factors or secretion of pro-dormant T-cell-originated cytokines, lack of outside-in β1 integrin signaling, triggered by anticancer therapy, especially tyrosine kinase inhibitors (TKI) (Endo and Inoue, 2019; Paget, 1889; Wang et al., 2019; White et al., 2004)	Myc suppression, mTOR suppression, and upregulated polycomb complex members (such as CBX7), leading to H3K27me3-marked gene repression, chemotherapy but not CDKi may evoke a diapause-like transcriptional expression profile (Dhimolea et al., 2021; Hu et al., 2020; Scognamiglio et al., 2016)
*(In)sensitivity to external growth stimuli*	Insensitive	Sensitive	Potentially sensitive	Sensitive
*Cell death sensitivity*	Reduced due to elevated anti-apoptotic pathways (Bcl2 family members, pro-survival kinase networks) (Zhu et al., 2017)	Variable	Insensitive (Bcl2 family members upregulated) (Minassian et al., 2019)	Low apoptotic priming (Dhimolea et al., 2021)
*Metabolic characteristics and autophagic state*	Hypermetabolic, active autophagy (also termed “geroconversion”) (Blagosklonny, 2014; Dörr et al., 2013; Kaplon et al., 2013; Young et al., 2009)	Decreased metabolic activity, enhanced autophagy and mitophagy (Marescal and Cheeseman, 2020)	Very low metabolic activity, minimized energetic (ATP) needs, active autophagy (Endo and Inoue, 2019)	Low metabolic activity, closely linked to activated autophagy (Dhimolea et al., 2021)
*Transcriptional and translational activity*	Enhanced, based on complex (de)regulation (Dörr et al., 2013)	Reduced biosynthesis	Reduced biosynthesis, “hypotranscription”	Profoundly reduced biosynthesis (Dhimolea et al., 2021; Scognamiglio et al., 2016)
*Epigenomic reorganization and cellular plasticity*	Extensive (Chandra et al., 2015; De Cecco et al., 2013; Martínez-Zamudio et al., 2020, 2023; Narita et al., 2006; Shah et al., 2013; Tasdemir et al., 2016; Zhang et al., 2005)	Remains to be investigated in greater detail, potential overlap with analyses from senescent and dormant cells	Remains to be investigated in greater detail, potential overlap with analyses from senescent and dormant cells	Remains to be investigated in greater detail
*Cell morphology*	Enlarged, flattened, vacuole/granule-rich, vanishing cell borders, SAHF, multi-nucleation (Dimri et al., 1995; Hayflick and Moorhead, 1961; Narita et al., 2003; Serrano et al., 1997)	Reduced cell size, potentially invasive and migrating (Triana-Martínez et al., 2020)	High migration capacity (Wnt-, RANK-dependent) (Triana-Martínez et al., 2020)	Not consistently reported yet
*Environmental remodeling and immune crosstalk*	SASP, exocytosis, cytoplasmic cell–cell bridges, immune recognition by innate and adaptive immune cells, upregulation of MHC I/II and immune checkpoint ligands (Chen et al., 2023a; Chuprin et al., 2013; Coppé et al., 2008; Eggert et al., 2016; Kang et al., 2011; Marin et al., 2023; Reimann et al., 2021; Sagiv et al., 2013; Xue et al., 2007)	No consistent reports	MHC II upregulated, but adaptive immune resistance (“immune cloaking”) via upregulation of immune checkpoint ligands, potentially SASP-like secretome (Phan and Croucher, 2020; Triana-Martínez et al., 2020)	No consistent reports
(*Ir*)*reversibility and underlying mechanisms*	Escape mostly via endogenous (epi)genetic defects, H3K9 demethylation, CDK inhibitor loss, Rb or p53 inactivation (Beauséjour et al., 2003; Lee and Schmitt, 2019; Martínez-Zamudio et al., 2023; Milanovic et al., 2018; Rane et al., 2002; Sage et al., 2003; Saleh et al., 2019; Schleich et al., 2020; Yu et al., 2018)	Reversible via extrinsic growth-promoting signals, e.g., through Coco, Noggin, Taz, FAK-ERK-Yap (Triana-Martínez et al., 2020)	Reversible via blockade of p38MAPK activity, but typically through extrinsic growth-promoting signals (Aguirre-Ghiso et al., 2003)	Reversible, potentially via Myc reelevation
*Functional fate upon arrest cessation*	Self-renewal, cancer stemness, reprogramming, plasticity/transdifferentiation, promotion of metastasis (Demaria et al., 2017; Laberge et al., 2012; Lapasset et al., 2011; Milanovic et al., 2018; Mosteiro et al., 2016; Ritschka et al., 2017; Webster et al., 2015)	Regrowth	Some similarity of dormancy and tissue stem cells, “awakening” into proliferation/self-renewal by growth factors and changes in niche conditions (Phan and Croucher, 2020)	Exit from diapause reinstates pluripotency, rather reestablishment of previous growth capacity when exiting from diapause-like conditions (Dhimolea et al., 2021; Scognamiglio et al., 2016)
*Therapeutic targeting*	Rather drug-resistant, but susceptible to senomorphics (to blunt the SASP) or senolytics (to selectively eliminate) (Birch and Gil, 2020; Chaib et al., 2022)	Rather drug-resistant, but susceptible to some targeted therapies or senolytics upon conversion to senescence (geroconversion) as a “lock-in” strategy, alternatively growth factor-enforced “lock-out” strategy followed by conventional anticancer agents (Marescal and Cheeseman, 2020; Triana-Martínez et al., 2020)	Rather drug-resistant, but susceptible to targeting of niche factors (e.g., CXCR4 antagonist, hypomethylating agents such as 5-azacytidine, proteasome blockade, G-CSF), Axl inhibition, YAP/TEAD targeting, potentially susceptible to senolytics with or without preceding (gero-)conversion to senescence (Kurppa et al., 2020; Phan and Croucher, 2020)	Rather drug-resistant, reminiscent of a TKI-preexposed “drug-tolerant persister” state, sensitive to CDK9 inhibition (Dhimolea et al., 2021; Hata et al., 2016; Rehman et al., 2021)
*Best discriminating markers*	SA-β-gal, high-level p16^INK4a^, H3K9me3, and—less discriminative—DDR signature, PML bodies, NF-κB and C/EBPβ activity, SASP, elevated urokinase-plasminogen activator receptor (uPAR) expression (Amor et al., 2020; Bartkova et al., 2006; Braig et al., 2005; Coppé et al., 2008; de Stanchina et al., 2004; Dimri et al., 1995; Kuilman et al., 2008; Serrano et al., 1997)	Not very distinctive, elevated CDKi such as p21^CIP1^ and p27^KIP1^, enhanced TGF-β, HIFα1 and Gas6 signaling (Triana-Martínez et al., 2020)	Low ERK/p38MAPK ratio, low Myc levels, low pAKT and mTORC1 signaling, increased NR2F1, SPARC, low uPAR expression, and—less discriminative—elevated TGF-β2 signaling, increased stemness (Wnt, Rank, Nanog, Sox9), enhanced endoplasmic reticulum stress (Aguirre Ghiso et al., 1999; Endo and Inoue, 2019; Phan and Croucher, 2020)	Low Myc levels, and—less discriminative—decreased mTOR signaling, activated ERK1/2 signaling

Of note, there is no clear genetics- or marker-based evidence that these conditions are biologically truly distinct principles; it remains conceivable that they present with largely overlapping but tissue- or context-dependent variations and may even reflect dynamically interchangeable presentations of the same cell over time.

a Including less clearly characterized states such as cellular hibernation or topor (Bouma et al., 2012; Dias et al., 2021; Oedekoven et al., 2021)

Senescent cells may not always exhibit all the features listed above. Therefore, it remains to be clarified which of them are mandatory for a cell to be considered as being senescent, and, hence, should stay positive during such a cell’s remaining life time, if the senescence status is truly irreversible. While most researchers probably understand the senescence-associated growth arrest as the hallmark of senescent cells, there is no broad consent on other senescence-associated facets beyond the SASP. In turn, cells displaying a full-featured senescence phenotype yet failing to firmly arrest may have converted DDR-driven upstream signals into senescence-typical epigenomic and cytoplasmic alterations. Such cells might exhibit a SASP as well, making a binary discrimination between “senescent or not,” including speculations on the potential reversibility virtually impossible. While there is a consensus on an urgent need to unify experimental activities, homogenize methodological approaches, and standardize read-outs in an unbiased manner within the senescence community, we are less convinced that a senescence definition would be widely agreeable and ready for prime-time. We feel that fundamental discoveries related to heterogeneity, dynamics, (ir)reversibility, immunogenicity and functional implications of the senescent state switch, uncertainties about its depth and the likely existence of incompletely featured subtypes during entry, and maintenance or exit from senescence ask for coordinated and accelerated research efforts. This includes single-cell-based atlas-like mapping activities that utilize curated marker gene sets across tissues and disease conditions rather than premature or even misleading definitions that may eventually “by definition” preclude pivotal novel insights from being acknowledged.

## Cleared physiologic versus persistent pathologic senescence

As alluded to, senescence serves as a physiologic rheostat in embryonic development and tissue homeostasis and reflects a normal cellular response to critically shortened telomeres during natural organismic aging. Key to all controlled implications of senescent cells is their transient presence since they get regularly cleared by innate and adaptive components of the host immune system or autonomously undergo secondary types of cell death (Chen et al., 2023a; Eggert et al., 2016; Kang et al., 2011; Marin et al., 2023; Reimann et al., 2021; Xue et al., 2007). In stark contrast, senescent cells that persist in the body for extended periods of time bear pathogenic potential. Senescent cells tend to accumulate in aged individuals due to their formation at elevated rates, which is due to replicative exhaustion and less accurate DNA repair. This accumulation is augmented by secondary paracrine or SASP factor-mediated senescence spreading to adjacent cells in aged environments with their enhanced loads of pre-senescent cells, further aggravated by insufficient clearance capacity of the elderly immune system (Schmitt et al., 2022). These persistent senescent cells with their chronic secretion of inflammatory and fibrogenic SASP factors, dubbed “inflammaging” (Franceschi et al., 2018), represent key contributors to pathologic aging. Age-related pathologies comprise, for instance, chronic pulmonary and hepatic fibrosis, COVID-19, diabetes, bone loss, osteoarthritis, sarcopenia, and neurodegeneration (McHugh and Gil, 2018; Schmitt et al., 2022; van Deursen, 2014). Accordingly, determining the quantity and quality of the (pre-)senescent cell burden during aging became a high-priority research objective. The cellular senescence network SenNet, an NIH-funded consortium, and other research groups seek to map senescent cells across numerous tissues throughout the human lifespan to characterize their role in physiologic aging (SenNet Consortium, 2022; Saul et al., 2022). Important additional insights were obtained by the selective pharmacologic removal of senescent cells, termed “senolysis.” Senolysis has been introduced as a prime therapeutic strategy to extend a healthy lifespan by delaying features of pathologic aging and to prevent detrimental implications related to long-term-arrested cancer cells (Baker et al., 2011, 2016; Dörr et al., 2013; Xu et al., 2018). Notably, the common denominator of all “good” or beneficial types of senescence is the limited presence of the respective cells—either by cell-autonomous ways like extrinsic immune cell-mediated clearance or via senolytic therapies—thereby indicating that the natural default of senescence is supposedly transient, not a lasting, and, thus, deleterious persistence of these cells.

## A matter of depth and quality

The view of senescence as an irreversible arrest is closely linked to its understanding as a binary condition—being in or not—thereby implying a certain threshold of cellular stress needed to execute the state switch. However, there is no clear evidence for such threshold, i.e., the accumulation of a critical amount of stress-induced cellular changes. Even if there were, it remains to be investigated whether an acute peak or the cumulative damage over time would be decisive. Senescence is not the uncoordinated result of a severe physical cellular insult but the consequence of a trigger-sensitive molecular program comprising effector cascades that control the entry and maintenance phase. Hence, quantitative and temporal aspects of these signals may not only determine when such putative threshold to senesce is reached but might further account for the quality and depth of the induced condition. In turn, an entry threshold to senescence would not necessarily reflect a point of no return nor the absence of any ongoing dynamic changes.

Given the phenotypic variations of senescence and senescence-like conditions reported in the literature, e.g., SA-β-gal reactivity, detectability of SAHF, morphological changes of cell body and nuclear size, amount and composition of the SASP, it seems appropriate to consider “nascent” (i.e., partial, incomplete, perhaps abortive) versus “fully established” (i.e., complete, non-abortive) forms of senescence on one hand and lighter versus deeper senescence states on the other hand. Different kinds of triggers, their duration at a certain amplitude or frequency, as well as cell type–specific contexts may all contribute to the quantitative and qualitative presentation of an agreeably full-featured senescence phenotype. For instance, in experiments using a tetracycline-inducible *H-Ras-G12V* allele allowing the titrated expression of oncogenic Ras at levels comparable with those of endogenous Ras or reflecting gross overexpression, OIS was only observed in response to supraphysiologic Ras-G12V expression in mammary epithelial cells. This supports the view that OIS detected in vivo might be restricted to locus amplification- or translocation-enhanced oncogene expression settings (Braig et al., 2005; Sarkisian et al., 2007). In other experimental settings, unlocking low-level oncogenic Ras expression from its endogenous alleles by recombining a transcriptional lox-stop-lox (LSL) cassette demonstrably resulted in OIS in the lung or the pancreas (Collado et al., 2005). Hence, quantitative aspects of prosenescent triggers certainly affect the concrete senescence read-out, with flanking cell-autonomous and non-cell-autonomous contexts operating as critical modulators not only of the senescence phenotype but potentially its lasting persistence as well.

## Active maintenance required

Cell populations that quantitatively entered a full-featured senescent growth arrest may remain lastingly arrested (Fumagalli et al., 2014) or reenter the cell cycle at some point. For example, Braf-V600E-driven melanoma cells in oncogene inhibition-senescence (i.e., a firm OIS-like condition due to acute cessation of “oncogene-addicted” signal dependence) resumed to divide upon removal of the Braf-V600E inhibitor vemurafenib (i.e., a post-senescence condition with yet-to-be-determined molecular underpinnings) (Haferkamp et al., 2013). Another example refers to TIS cells in which senescence-essential Suv39h1 or p53 gene products were only transiently expressed, permitting reproliferation out of senescence as soon as their expression levels became critically low (Milanovic et al., 2018). One may argue that seemingly senescent cells that restarted proliferation were in fact never senescent. However, many biological processes without structural destruction, like enzymatic reactions, self-renewal properties, or immune cell activation and subsequent inactivation, are reversible (Chaffer et al., 2011; Sun et al., 2023). Biological activities or state switches, once induced by appropriate triggers, are typically not passive endpoints but rather rely on active maintenance mechanisms. Experimental evidence in this regard came from investigations that addressed nucleosome turnover, i.e., the need to reestablish senescence-associated histone marks upon scheduled exchange of K9-trimethylated histone H3 by newly synthesized non-methylated H3 (see Fig. 1 B). An experiment using a non-methylatable mutant histone H3.1 (i.e., H3R9) demonstrated that senescence-associated histone methylation marks (H3K9me3) can be progressively replaced by this mutant in senescent cells. Unlike the H3.3 variant, H3.1 deposition into nucleosomes is strictly replication-coupled (Ahmad and Henikoff, 2002; Yu et al., 2018). This approach provided two important insights: first, senescent cells, despite their non-dividing state, keep trying to replicate DNA, thereby incorporating newly synthesized histones into nucleosomes, albeit in a futile manner that rapidly ends at stalled replication forks before undertaking a next unsuccessful attempt. Second, the incorporation of the artificial non-K9-methylatable H3R9 mutant progressively replaced senescence-essential H3K9me3, and, thus, licensed an ultimate exit from senescence (Yu et al., 2018). Accordingly, senescence relies on continuously reinstated K9 trimethylation to retain the cell in senescence (see Fig. 1 B regarding related control mechanisms).

Further underscoring the dynamic nature of senescent cells, investigations over extended periods of time increasingly pinpoint (epi)genomic reorganization or modulated composition of SASP waves in the course of senescence establishment and the subsequent maintenance phase (Chandra et al., 2015; Hoare et al., 2016; Kolesnichenko et al., 2021; Sadaie et al., 2013; Shah et al., 2013). Unlike the apoptotic cascade with its ultimately destructive and cytolytic events exerted by proteases and nucleases, senescence comes with rather subtle, albeit potentially critical cellular damage—such as the continuous depletion of lamin B1 from the nuclear envelope with consecutive effects on genomic H3K9me3 redistribution and release of heterochromatin into the cytosol (Ivanov et al., 2013; Sadaie et al., 2013; Shah et al., 2013). These cytoplasmic chromatin fragments were shown to drive cGAS/STING signaling, thereby triggering an interferon response, enhancing the SASP, and reinforcing cell-autonomous and paracrine senescence (Dou et al., 2017; Glück et al., 2017; Gulen et al., 2023; Ivanov et al., 2013). Presumably, the continuous loss of nuclear material will no longer be compatible with further cell survival at some point. Another feature of senescent cells is the background oxidative stress due to a lowered NAD^+^/NADH ratio or glutathione depletion (Igelmann et al., 2021; Muller, 2009; Ngoi et al., 2021; Sun et al., 2018), thereby exposing the massive amount of SASP and other proteins to premature oxidization and misfolding. This proteo-stress condition ultimately creates a metabolic dependency on ATP-providing pathways to fuel energy-consuming protein degradation and autophagic disposal of the toxic peptides. These actions are needed to prevent an unfolded protein response with endoplasmic reticulum-associated protein degradation and, potentially, subsequent cell death (Dörr et al., 2013). In essence, senescent cells exhibit highly active metabolic features that accommodate cell-intrinsic needs, especially to cope with survival-threatening toxicities. Such metabolic changes reflect adaptations to external conditions and indicate complex biochemical maintenance machinery required by senescent cells to lastingly persist as such. If essential senescence maintenance is interrupted, cells may die or survive as senescence escapees (Fig. 1 C).

## No reversal but post-senescence

Questioning the lasting stability of a senescent cell-cycle arrest led to speculations about the potential reversibility of the senescent phenotype—suggesting a mechanism by which regrowing senescent cells might reestablish a cellular condition indistinguishable from the pre-senescent state. In this overview, we like to emphasize our slightly different perception of this problem: given the profound chromatin remodeling cells undergo when entering and staying in senescence, it appears highly unlikely that a proliferation-permitting exit mode would fully and selectively revert the broad senescence-associated epigenomic alterations to the pre-senescent state (Fig. 1 C). We favor the view that proliferation reenabled previously senescent cells rather present as lastingly distinct from the very same population of cells that never entered senescence, leaving them with a “senescence scar,” a senescence-associated chromatin mark that remains detectable as an epigenetic memory in post-senescent cancer cells (Martínez-Zamudio et al., 2023). Recently, extensive epigenomic profiling identified AP-1 TF as top-hierarchy pioneers that interact with enhancers in an OIS-typical manner (Martínez-Zamudio et al., 2020). AP-1 not only orchestrates senescence entry but subsequently promotes senescence escape by facilitating interactions of senescence-induced TF such as POU2F2/Oct2 with enhancer chromatin (Martínez-Zamudio et al., 2023). Hence, probabilistic priming for TF binding in precoded enhancer landscapes underlies dynamic enhancer remodeling, drives senescence exit-enforcing transcriptional programs, and leaves chromatin scars as an epigenetic post-OIS memory behind. Collectively, this evidence strongly supports the view that senescence is not irreversible, and progression out of it is not reversible, and progression out of it is no reversal back to pre-senescence.

The output of such a scar might be persistent high-level p16^INK4a^ expression that was induced during senescence. For instance, p16 stays high in cervical cancer upon HPV E6-mediated inactivation of p53 function or lymphomas that overcame OIS or reprogressed out of TIS due to loss of intact *p53* alleles, irrespective of p16’s ability to inhibit CDK4 and CDK6 (Carozzi et al., 2008; Fu et al., 2003; Sandhu et al., 2000; Schmitt et al., 2002; Braig et al., 2005). Even more intriguing, we and others found senescent cells to undergo epigenomic reprogramming with a latent stem-like gene expression profile (Benítez et al., 2021; Martínez-Zamudio et al., 2023; Milanovic et al., 2018; Yu et al., 2023) that appeared to be retained by a small but constant fraction of post-senescent cells, executing their transcriptional remodeling as particularly aggressive tumor reinitiating cancer stem cells (Batlle and Clevers, 2017). As a consequence, senescence-associated stemness (SAS), especially strongly elevated Wnt signaling due to a much more expression-permissive chromatin environment in the surroundings of Wnt mediator loci, contributed to treatment failure in aggressive B cell lymphoma as well as de novo stemness in acute leukemia models in vivo (Milanovic et al., 2018). When senescent cells restart proliferation and exert their SAS, other senescence-associated features such as the SASP, metabolic rewiring, and enhanced plasticity, as well as increased immunogenicity may potentially be retained in post-senescent cells (Fig. 1 C). Consistent with this view, senescent scars with upregulated POU2F2/Oct2 chromatin binding activity were found in colorectal cancer patients who progressed with a particular detrimental, SASP-reminiscent inflammatory biology (Martínez-Zamudio et al., 2023).

Conversely, cells that exited from senescence in the course of dynamic enhancer remodeling may potentially later reenter senescence, as preliminary evidence with switchable gene moieties implied (Milanovic et al., 2018). This is of clinical importance when considering the prosenescent pressure of anticancer therapies in tumors that formed as OIS-overcoming, post-senescent malignancies. In the absence of senescence-compromising structural gene defects, one could envision senescence entry and exit in a bidirectional fashion based on the predominant senescence-promoting or -antagonizing molecular setting in a given cell at a given time. Within the stochastics of state-critical molecular interactions over time, it is conceivable that some cells may actually oscillate between a senescent and a non-senescent state—reminiscent of the probabilistic determination of state switches and overlap conditions in between, described by quantum physics (Heisenberg, 1927; Schrödinger, 1935; Wheat et al., 2020). Increasing scientific interest in liquid–liquid phase separation highlights the role of non-membrane-compartmentalized macromolecules in executing biochemical reactions in the right concentration at the right place and moment. Examples like 53BP1-related senescence-associated heterochromatinization, PML bodies forming as stress-inducible nuclear condensates, or cytoplasmic cGAS/STING stress granule assembly emerge in time and space by liquid–liquid phase separation and illustrate the probabilistic nature that underlies components of the senescent state switch and its persistence (Alberti et al., 2019; Igelmann et al., 2021; Liebl and Hofmann, 2022; Zhang et al., 2022).

## A senescent state-switch of already firmly arrested cells

Additional clues to senescence occurring as an arrest-unrelated condition came from post-mitotic “G0” cells. In certain settings, these cells exhibit a senescent phenotype in response to stress, irrespective of the stably arrested growth condition. In age-related macular degeneration (AMD), for instance, the virtually non-proliferating retinal pigment epithelial cells get activated and slowly divide, upon which senescence induction becomes a critical pathogenic step toward AMD (Markovets et al., 2011; Stern and Temple, 2015). In a broader sense, neurodegenerative diseases such as Alzheimer’s disease have also been linked to the senescence of post-mitotic neurons (Bussian et al., 2018; Herdy et al., 2022). A key driver of post-mitotic senescence is mitochondrial dysfunction, which promotes pro-senescent metabolic changes and the generation of reactive oxygen species (ROS) in these non-dividing cells (Wiley et al., 2016). In turn, ROS may cause difficult-to-repair damage at telomeres as well as telomere length-independent senescence in post-mitotic cells, as reported for cardiomyocytes and other cell types (Anderson et al., 2019; Fumagalli et al., 2012; Hewitt et al., 2012). We speculate that senescence induction in post-mitotic cells primarily occurs to engage in environmental crosstalk and inflammatory presentation to the immune system—with a hard-to-predict beneficial or detrimental outcome. Another reason might be senescence-associated plasticity, equipping post-mitotic cells with novel cell-autonomous functionalities to better cope with the encountered stresses. Whether post-mitotic senescent cells will ever progress to a post-senescent, proliferation-reenabled phase and which of the (not directly arrest-controlling) senescence-associated features may be retained remain to be investigated. In essence, the dynamics of cellular senescence and their molecular underpinnings in various subcellular compartments with the programmatic and deterministic, or rather stochastic and uncertain execution of subsequent changes, are key to the long-term stability of the senescent state.

## Overriding senescence: Escape versus bypass

If OIS serves as a critical barrier to full-blown tumor development and TIS is an important effector program of anticancer therapies, senescence-disabling molecular mechanisms should be selected under these conditions. We and others demonstrated this for loci such as *p53* or *INK4a/ARF* (i.e., *CDKN2A*), the p16^INK4a^-overriding CDK4-R24C moiety, the Rb protein family, as well as for compromised H3K9 trimethylation under the respected stresses in vivo (Beauséjour et al., 2003; Braig et al., 2005; Rane et al., 2002; Reimann et al., 2010; Sage et al., 2003; Schleich et al., 2020; Schmitt et al., 2002; Wölfel et al., 1995; Yu et al., 2018) (Fig. 1). Although terms like “overriding” or “disabling” senescence are frequently used in the literature, their actual meaning—whether to prevent senescence from occurring or to promote cell-cycle reentry out of senescence—is often poorly defined or even mixed-up with true escape when factually only bypass was experimentally addressed. Senescence control has been a target of extensive screening efforts based on genome-wide or focused complementary DNA, small-hairpin RNA, CRISPR, as well as pharmacological compound libraries (Acosta et al., 2013; Han et al., 2018; Innes et al., 2021; Liu et al., 2019; Peeper et al., 2002). Virtually all of the published screens so far were set up to track senescence bypass or modulation of senescence-associated features such as SASP profiles, not to identify gene activities or drug targets relevant for senescence escape. Here, we would like to emphasize that the biological consequences of encountering senescence-associated epigenomic remodeling and subsequently exiting senescence as a post-senescent cell would be fundamentally different from a cell in which no senescence-associated changes occurred in response to the same pro-senescent trigger (Fig. 1 C).

Senescence escape was first demonstrated as the result of acute loss of all *Rb* isoforms or activation of a dominant-negative p53 moiety in settings of manifest senescence (Beauséjour et al., 2003; Sage et al., 2003). Utilization of senescence-controlling genes, especially as regulatable versions thereof (e.g., *p53*, *Suv39h1*, *CDK4*, *JMJD2C*, *H3R9*, or *Myc* [Box 2; and Fig. 1, A and B]) (Hydbring et al., 2010; Milanovic et al., 2018; Rane et al., 2002; Ruggero et al., 2004; Yu et al., 2018), provided increasing support for the hypothesis that fully senescent cells may indeed get back into cycle upon activation of senescence-disabling moieties or critically reduced expression of essential senescence maintenance genes. Conversely, cells that would normally senesce in response to oncogenic Ras or Braf exhibited senescence bypass if they were a priori depleted of senescence-essential loci such as *p53* or *INK4a/ARF* (Serrano et al., 1997) or presented with overexpression of the H3K9 demethylase JMJD2C at the outset (Yu et al., 2018). Importantly, a fusion protein of JMJD2C and a 4-OH-tamoxifen (4-OHT)-inducible estrogen receptor (JMJD2C:ER^TAM^) (Littlewood et al., 1995) was able to drive senescence escape upon 4-OHT administration in full-featured OIS (Yu et al., 2018). Likewise, inducing loss of Suv39h1 or p53 expression by 4-OHT deprivation in cells engineered to produce the corresponding ER^TAM^ fusion proteins on a *suv39h1*-deficient or *p53*-null background also permitted cell-cycle reentry from senescence (Milanovic et al., 2018). Beyond enforcing senescence escape by interference with defined components of its maintenance machinery, we also tracked spontaneous DNA reduplication as an early indicator of resumed proliferation in cell models that had robustly entered senescence. Following a fluorescence-based vital SA-β-gal stain first, the transient codetection of a secondary fluorescent signal that labeled ongoing DNA synthesis was used as a unique marker tandem to catch the pivotal moment in individual senescent cells when they spontaneously exited their terminal arrest condition (Milanovic et al., 2018).

Box 2 Senescence maintenance-essential or senescence exit-conferring genes

• p53, loss of or dominant-negative mutant (Beauséjour et al., 2003; Milanovic et al., 2018; Serrano et al., 1997).
• Rb, collectively loss of all three isoforms, i.e., Rb, p107 and p130 (Sage et al., 2003).
• H3K9me3 histone methyltransferase Suv39h1, loss of (in murine B-lymphocytes) (Braig et al., 2005; Milanovic et al., 2018).
• CDK4, overexpression of the p16^INK4a^-insensitive mutant CDK4-R24C (Rane et al., 2002).
• Myc, cooperation with OIS-enforcing oncogenic Ras (Hydbring et al., 2010; Land et al., 1983; Ruggero et al., 2004).
• H3K9-active demethylase JMJD2C (Yu et al., 2018).
• H3K9-active demethylase LSD1 (Yu et al., 2018).

Despite accumulating evidence for such an exit, only fate-tracking of individual cells can robustly underscore that deep cellular senescence is not necessarily a non-proliferative endpoint of a cell’s lifetime. Reporter mice carrying an *INK4a* promoter-driven Cre:ER^TAM^ recombinase can be used to indirectly mark cells in senescence with high-level activation of p16^INK4a^, both in vitro and in vivo. In this model, inducible Cre will unlock the expression of a reporter by deleting the stop codon in front of the fluorescence coding sequence. Subsequently redividing cells will retain a fluorescent “scar” indicative of their previous senescence state (Omori et al., 2020). Moreover, CRISPRa tracing of clones in heterogeneous cell populations (CaTCH) or other similar technological advances (Li et al., 2023; Umkehrer et al., 2021) enable fate-tracking of individual subclones during their journey into and out of senescence. Coupling such barcoding technique with senescence scar-reporting would create a very powerful and trustable setting suitable for demonstrating spontaneous exit from bona fide senescence and investigating its functional distinctions from cells that never entered senescence.

## A wall to reprogramming

The stable senescent cell-cycle arrest was long considered a barrier to induced pluripotency. The two pivotal senescence-co-controlling gene loci *p53* and *INK4a/ARF* were reported to limit the efficacy of induced pluripotent stem cell (iPSC) generation by launching a DDR and promoting subsequent apoptosis, while a priori ablation of p53 or p16^INK4a^/ARF expression enhanced reprogramming (Banito et al., 2009; Kawamura et al., 2009; Li et al., 2009; Marión et al., 2009; Utikal et al., 2009). Notably, methylation-based silencing of the *INK4a/ARF* locus was found to occur as a late event in settings with superior reprogramming capacity, suggesting a dynamic contribution of senescence control in this context (Li et al., 2009; Utikal et al., 2009). The epigenetic mode of inactivation would not require DNA replication; hence, would permit loss of expression during manifest senescence. When the OSKM reprogramming factors (i.e., Oct4, Sox2, Klf4, and c-Myc) were extended by Nanog and Lin28, senescent cells were readily reprogrammable and presented with a full reversion of their age-related phenotypes (Lapasset et al., 2011). Consistent with this, successful chemical reprogramming out of a diapause-like state by a cocktail of small compounds was just reported (Chen et al., 2023b). Moreover, the recent observation that POU2F2/Oct2 can facilitate exit from OIS further supports a dynamic senescence-in/senescence-out sequence needed to fully unleash the contribution of senescence to efficient reprogramming (Martínez-Zamudio et al., 2023). Interestingly, investigations employing a mouse model with an inducible OSKM transgene unveiled strong cooperativity of stress-related senescence evoked by OSKM factors, age, and tissue injury toward enhanced reprogramming efficacy in vivo (Mosteiro et al., 2016). This was recently further supported by the observation of a senescence-reprogramming link in cnidarian and axolotl during tissue regeneration (Salinas-Saavedra et al., 2023; Yu et al., 2023).

**Figure 2.**
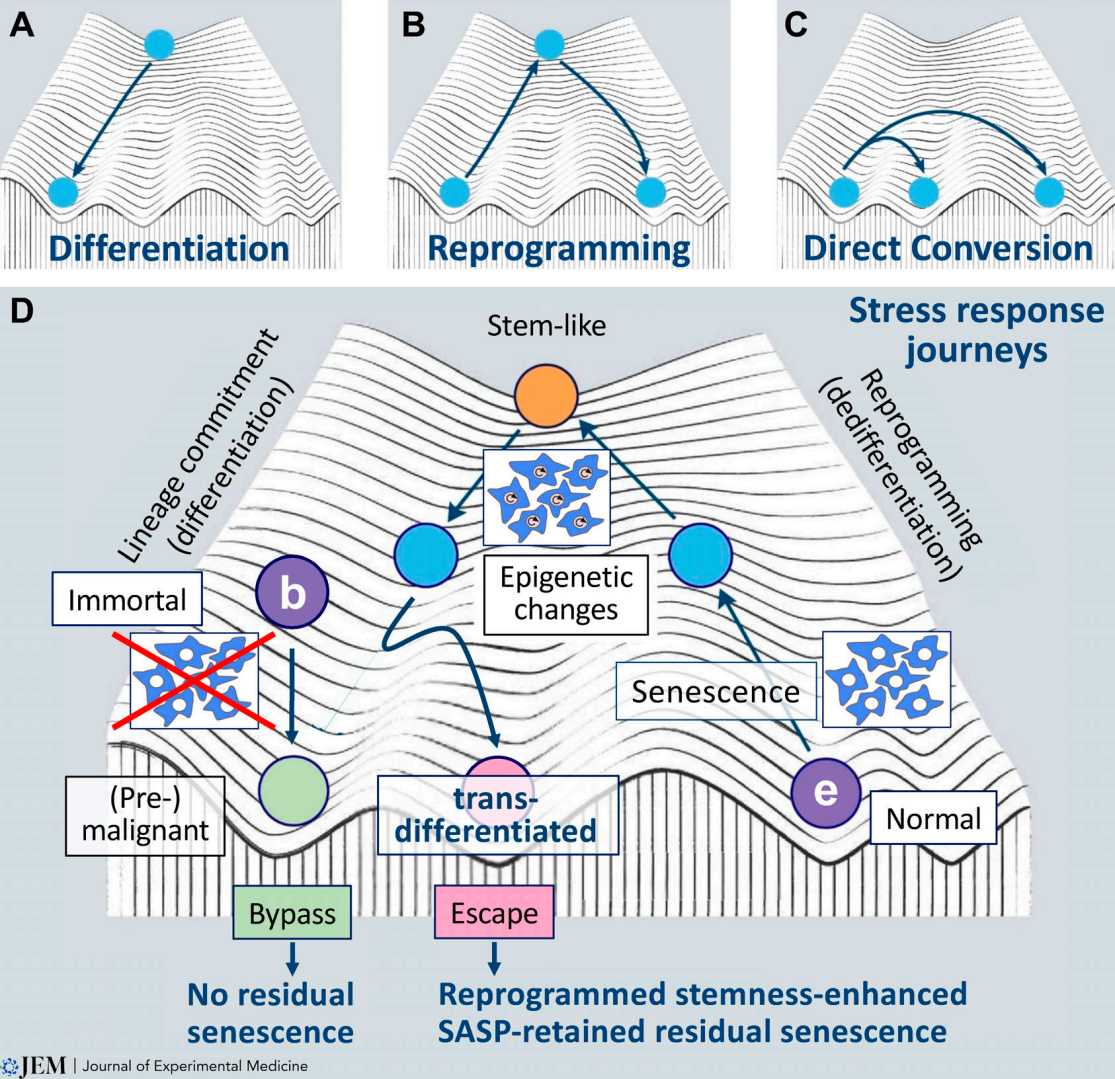
**Transient senescence passaging during stress response journeys alters cell fate.** Epigenetic remodeling and chromatin dynamics shape a Waddington-reminiscent landscape of senescence-associated stemness and plasticity. **(A–C)** Fate models refer to normal differentiation from a progenitor (A), iPSC-like pluripotent reprogramming (B), and direct conversion from one to another terminally differentiated cell type (C). **(D)** Stress response journeys (bold arrows) of senescence-incapable (immortal) cells that bypass (b) cellular senescence upon oncogenic stress exposure on their path to a (pre-)malignant state as compared with normal cells transiently encountering oncogenic stress-induced senescence and undergoing profound epigenetic changes including stem-like reprogramming and altered lineage commitment (i.e., phenotypic plasticity) before ultimately escaping (e) from the arrest as post-senescent cells. The profound senescence-related epigenetic changes determine a distinctly different cell fate, as depicted by the transdifferentiated cellular offspring now found in a different Waddington valley (adapted from Takahashi and Yamanaka, 2016; Waddington, 1957).

Epigenomic remodeling links senescence to phenotypic plasticity (Laberge et al., 2012; Liu et al., 2008; Ritschka et al., 2017). Supported by additional studies from others (Benítez et al., 2021; Ritschka et al., 2017; Saleh et al., 2019), we found senescence-related cell-autonomous remodeling to underly acquired self-renewal capacity and epigenetic plasticity (Milanovic et al., 2018), which is needed for proper, full-featured tissue regeneration after injury (Demaria et al., 2014; Lee and Schmitt, 2019; Ritschka et al., 2017). Regulatable switches of senescence-essential gene moieties allowed us to control entry into and exit from senescence. Stem-like and lineage-promiscuous transcriptional changes occurred during senescence establishment but remained functionally latent in these non-dividing cells—until post-senescent cells with regained proliferative capacity phenotypically executed these stemness properties. In essence, senescence represents both a barrier to and a promoter of reprogramming, as illustrated by senescence-enabled stemness and transdifferentiation in a Waddington-reminiscent landscape model (Fig. 2, adapted from Takahashi and Yamanaka, 2016; Waddington, 1957). In such a model, the initial cell state is depicted as a protected valley, in which senescence-associated epigenetic changes occur that will become phenotypically fully evident only upon subsequent release from the senescent cell-cycle block, i.e., by overcoming the adjacent wall toward the next valley, reflecting a fundamentally different cell fate. Notably, it is currently unclear whether senescence-associated epigenetic plasticity may lead to a complete or only a partial conversion to other cell types either within a tissue, e.g., from mature B cells to macrophages within the hematopoietic system (Xie et al., 2004) or even to different tissues of origin, e.g., mucosa cells to fibroblasts during epithelial–mesenchymal transition (Ansieau et al., 2008). It is also uncertain whether such plasticity might occur as direct conversion independently of a dedifferentiation step via pluripotency reprogramming. It will be interesting to see whether the classic dogma of pluripotency versus differentiation as incompatible states within one given cell is respected by the senescent condition—or whether stem-like capability and aberrant differentiation might coexist there (McKenzie et al., 2019; Metcalf, 1999).

## An operational ecosystem

Due to the lack of robust senescence-determining markers and their partly limited in vivo applicability (Gorgoulis et al., 2019), the role of senescence in disease and therapy remains understudied, especially in tissue-wide or organismic contexts. Given the interactivity and dynamics of the senescent condition—underscored by a large body of work related to SASP, secondary senescence of adjacent stromal cells, and immunogenicity as non-cell-autonomous ramifications of senescence (Acosta et al., 2013; Chen et al., 2023a; Coppé et al., 2008; Eggert et al., 2016; Hoare et al., 2016; Kang et al., 2011; Kolesnichenko et al., 2021; Krtolica et al., 2001; Lee et al., 2021; Marin et al., 2023; Reimann et al., 2010; Reimann et al., 2021; Xue et al., 2007)—it is particularly relevant to investigate how senescent cells operate as components within their “ecosystems” over time, a microenvironment they actively shape to some degree. State-of-the-art spatial single-cell transcript and protein analyses will provide unparalleled insights into the functional interactions of senescent cells with surrounding cells (Karimi et al., 2023; Sorin et al., 2023). On the organismic level, well-characterized, genetically tractable animal models of senescence-related diseases such as cancer, fibrotic pulmonary disease, COVID-19, or osteoarthritis (Dörr et al., 2013; Jeon et al., 2017; Lee et al., 2021; Omori et al., 2020; Reimann et al., 2021; Schafer et al., 2017; Schleich et al., 2020; Schmitt et al., 2002), for instance, are increasingly exploitable by proliferation-related PET imaging and fate-tracking of senescent cells in vivo (Dörr et al., 2013; Milanovic et al., 2018; Schleich et al., 2020; Umkehrer et al., 2021; Xu et al., 2017). These will be instrumental for deciphering the long-term beneficial or detrimental corollaries cellular senescence and senolytic therapies have in these complex biological processes in whole organisms (Box 2). Together, those models in conjunction with primary patient material will provide unparalleled insights into how senescent cells impinge on tissue functionalities over time, especially when serial intraindividual biopsies prior to and after senescence-relevant interventions are available.

Such system-wide analyses will address three central aspects of the senescent state switch: dynamics, heterogeneity, and quantitative impact. Molecular control and related dynamics of senescence stability, turnaround of key mediators, and composition of the associated secretome were touched on before (see above). Obviously, multicellular interdependencies of senescent cells with adjacent stromal and immune cells, as well as the induction of paracrine senescence in surrounding cells, including mobile elements such as macrophages, can spread local senescence state switches to distant sites in the body and impact organismic fate. This has been recently demonstrated for severe COVID-19 (Camell et al., 2021; D’Agnillo et al., 2021; Evangelou et al., 2022; Lee et al., 2021; Tsuji et al., 2022; Wang et al., 2021). Senescence heterogeneity and its quantitative ramifications remain largely understudied in the field. Senescence detectability is based on weak and loosely defined criteria, guided by the gold-standard SA-β-gal staining assay, which, however, fails to mark every cell even under homogeneous senescence conditions in vitro (Dimri et al., 1995). There is great uncertainty whether the remaining, non-stained cells might potentially turn positive shortly later, perhaps after a higher pro-senescent trigger dose, or never. How homogeneous or heterogeneous senescence responses actually are, and which biological consequences to expect therefrom remains to be investigated in much greater detail (Mahmoudi et al., 2019). Considering quantitative aspects, it certainly makes a huge difference whether protection from full-blown tumor development would rely on the robust proliferation block in nearly every cell of a multicellular Braf-V600E-driven melanocytic nevus, whether senescence-primed immunity would require a critical number or density of highly immunogenic cells to launch a broadly cytolytic adaptive immune response, or whether just a few senescent persister cells that occasionally managed to reenter the cell-cycle could act as de novo cancer stem cells and relapse drivers, while the majority of other senescent cells remain deeply arrested for good (Chen et al., 2023a; Marin et al., 2023; Milanovic et al., 2018; Moiseeva et al., 2023). In non-malignant settings, especially age-related pathologies, the overall and cumulative burden of senescent cells is probably much more important, as their fibrogenic and inflammatory potential is presumably quite proportional to their organismic or organ-specific load (Franceschi et al., 2018).

While meaningful and comprehensive answers to these problems might be difficult to provide, interrogating ecosystems-embedded senescence by inducible genetic tools or pharmacological intervention is an informative and therapeutically relevant strategy. Specifically, interference with the SASP or selective elimination of senescent cells by senomorphic or senolytic interventions will help elucidate the functional contribution of senescent cells to pathogeneses and treatment outcomes over time (Birch and Gil, 2020; Chaib et al., 2022; Schmitt et al., 2023). Accordingly, the decision for senomorphic treatments to blunt the SASP or senolytic measurements to eliminate senescent cells—either as the source of SASP or because of their built-in risk to reenter the cell-cycle and exert cancer stem cell properties—might be context dependent. To determine the preferred strategy in a given context, clinical trials are needed to convert preclinical experiments into robust clinical evidence (Dörr et al., 2013; Gonzales et al., 2023; Hickson et al., 2019; Lee et al., 2021; Milanovic et al., 2018; Schmitt et al., 2023).

## Concluding remarks

Cellular senescence is neither irreversible nor reversible. Despite its prime presentation as a long-term stable proliferative arrest, senescence is a highly dynamic state that requires continuous maintenance mechanisms not to transition to a post-senescent condition—which appears to be fundamentally different from a similarly stressed but never-senescent cell population. By enabling secondary cell death out of senescence and active immune mechanisms to clear senescent cells, nature apparently did not intend to keep senescent cells for good. Presumably, the type and strength of senescence-enforcing triggers account for the depth and quality of senescence as a multifaceted phenotype well beyond a mere cell-cycle arrest. Molecular control of the senescent state switch might not always be equally stringent: partially executed epigenetic remodeling may result in less stable (but possibly still full-featured) “senescence light,” while deeply senescent cells may hardly reenter the cycle, even if upstream maintenance signaling is interrupted. The medical importance of these yet-to-be-elucidated aspects is manifold, as they underly aging, tumor development and recurrence (auto)immunity, neurodegeneration, and other non-malignant diseases. These involve qualitatively and quantitatively distinct cell-autonomous and non-cell-autonomous molecular mechanisms, which, therefore, seem to require tailored strategies for the most effective intervention.
